# Illness Perceptions are Associated with Quality of Life in Patients with Fibrous Dysplasia

**DOI:** 10.1007/s00223-017-0329-5

**Published:** 2017-10-11

**Authors:** B. C. J. Majoor, C. D. Andela, C. R. Quispel, M. Rotman, P. D. S. Dijkstra, N. A. T. Hamdy, A. A. Kaptein, N. M. Appelman-Dijkstra

**Affiliations:** 1 Department of Orthopaedic Surgery, Center for Bone Quality, Leiden University Medical Center, Albinusdreef 2, Postzone J11, PO Box 9600, 2300 RC Leiden, The Netherlands; 2 Division Endocrinology, Department of Medicine, Center for Bone Quality, Leiden University Medical Center, Leiden, The Netherlands; 3 Department of Medical Psychology, Center for Bone Quality, Leiden University Medical Center, Leiden, The Netherlands

**Keywords:** Fibrous dysplasia, McCune–Albright syndrome, Illness perceptions, Quality of Life, Self-management

## Abstract

Fibrous dysplasia (FD) is a rare bone disorder in which normal bone is replaced by fibrous tissue resulting in pain, deformities, pathological fractures or asymptomatic disease. Illness perceptions are patients’ cognitions and emotions about their illness and its treatment, which may impact on Quality of Life (QoL). Here, we explore illness perceptions in patients with FD compared to other disorders, identify factors associated with illness perceptions and evaluate their relationship with QoL. Ninety-seven out of 138 eligible patients from the LUMC FD cohort completed the Illness Perception Questionnaire-Revised (IPQ-R) and the Short Form-36 (SF-36). Age, Gender, Skeletal Burden Score (SBS), FGF-23 levels, type of FD and SF-36 scores were analysed for an association with illness perceptions. We observed significant (*p* < 0.01) differences in patients’ illness perceptions between FD subtypes in the domains: identity, timeline acute/chronic and consequences. Patients with craniofacial FD reported to perceive more consequences (*p* = 0.022). High SBS was associated with perceiving more negative consequences and attributing the cause of FD to psychological factors (*p* < 0.01), and high FGF-23 levels with attributing more symptoms to the disease and perceiving more consequences (*p* < 0.01). The IPQ-R domain identity, timeline acute/chronic, timeline cyclical, consequences, emotional representations and treatment control were significantly associated with impairments in QoL. Illness perceptions in patients with FD relate to QoL, differ from those in patients with other disorders, and are associated with disease severity. Identifying and addressing maladaptive illness perceptions may improve quality of life in patients with FD.

**Electronic supplementary material:**

The online version of this article (doi:10.1007/s00223-017-0329-5) contains supplementary material, which is available to authorized users.

## Introduction

Fibrous dysplasia (FD) is a rare bone disorder that is caused by a missense mutation of the GNAS gene, leading to improper function of the alpha subunit of the heterotrimeric G protein (G_s_α) that ultimately results in a local disorder of bone formation [1]. This heterogeneous disease can present with a wide spectrum of clinical manifestations from a lesion in a single bone (monostotic FD), in multiple bones (polyostotic FD) with or without endocrinopathies such as precocious puberty, or growth hormone excess in the McCune–Albright syndrome (MAS). FD lesions may be associated with bone pain, skeletal deformities and pathological fractures, although they may also be asymptomatic. Quality of Life (QoL) has been reported to be more severely affected in patients with the more severe types of FD. Determinants of severe disease including a High Skeletal Burden Score (SBS) and increased bone turnover markers (BTM), have been identified as determinants of impaired QoL [2, 3]. Other factors than those directly related to the disease which also influence QoL are ‘illness perceptions’: the thoughts and emotions that a patient has concerning his or her disease and its treatment [4]. These perceptions originate from various sources, such as information from social circles the patient moves in, information from health care providers, experiencing other or previous illnesses, information from other patients with the same disorder, or general or social media [5, 6]. The common-sense model (CSM) of self-regulation, which outlines illness perceptions and their relations with sociodemographic and clinical characteristics, and coping and outcome (QOL), aims at examining cognitive and emotional representations to illness [7, 8]. The CSM is based on the premise that an individual solves a problem (i.e., a perceived threat to health, an illness) in an active manner by trying to make sense of the threat to his or her health or illness. These perceptions are clustered around five components, forming the illness perceptions that determine the patients’ coping behavior. These cognitive components are (1) *identity* the label that the individual uses to describe the condition and its symptoms, (2) *cause* ideas that the individual has about the cause(s) of the condition, (3) *timeline* expectations of the individual about the duration of the condition, (4) *consequences* effects of the disease on the physical, psychological and social functioning of the individual, and (5) *cure/control* the extent to which the individual perceives that the condition is amenable to cure and/or control [9].

The Illness Perception Questionnaire-Revised measures these illness perceptions in patients with different diseases [10, 11]. Since illness perceptions have been shown to influence QoL, it is important to characterize these perceptions in patients with FD [12]. In this study, we explore illness perceptions in patients with FD, as an extension of a previous study evaluating QoL in the same population [3]. We further explore the relationship between illness perceptions and QoL in patients with FD and also aim at identifying associated factors for maladaptive illness perceptions.

## Subjects and Methods

### Patients (Fig. 1)

All patients aged 15 years or older and who had been seen at the outpatient clinic of the LUMC in the previous 3 years were asked to take part in the study. Patients were invited by mail to participate in the study, which implied filling in a number of questionnaires, including the Illness Perception Questionnaire-Revised (IPQ-R) and the Short Form 36 (SF-36), a questionnaire assessing functional status, as previously described [3]. Patients who did not respond were approached by telephone with a maximum of two attempts.

**Fig. 1:**
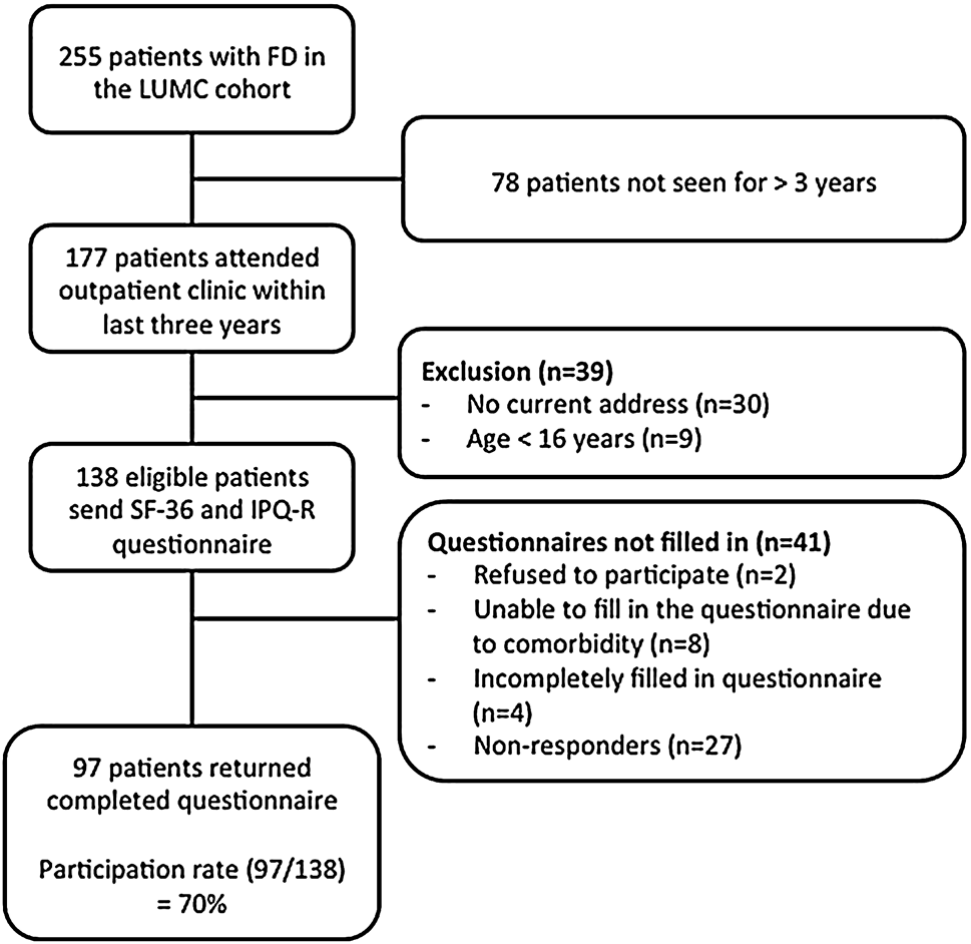
Flowchart of patient inclusion and participation rate

### Clinical and Sociodemographic Characteristics

Patient data were retrieved from electronic medical records. Level of education was categorized using the International Standard Classification of Education (ISCED) [3]. Low level of education was defined as a primary to lower secondary education; medium level of education was defined as an upper secondary to post-secondary non-tertiary education; and high level of education was defined as the first and second stage of tertiary education. Data on age, gender, age at diagnosis, type of FD, fractures and extraskeletal manifestations (e.g., precocious puberty, GH-excess, café-au-lait patches) were collected. Data on medical or surgical treatment were also retrieved from medical records. Fibroblast growth factor 23 (FGF-23) was obtained from serum measurements within 2 months of completing the questionnaires [13]. Skeletal burden scores (SBS) were blindly assessed by two authors (BCJM and NMA-D) for all patients in whom a ^99^Technetium skeletal scintigraphy was available [14].

### Illness Perception Questionnaire-Revised (IPQ-R)

The IPQ-R questionnaire was designed to evaluate cognitive and emotional representations of illness in patients with chronic or acute conditions [10, 11]. The questionnaire consists of three different parts: illness identity, illness perception and causal attributions.

#### Identity

This part of the questionnaire is built-up of 14 commonly occurring symptoms and of 13 commonly occurring symptoms related to the disease under study, in this case FD. Patients are asked if they have any of these 27 symptoms and whether they believe that their disease is the cause of these symptoms. The score on the *identity* subscale represents the degree to which patients relate common symptoms they suffer from to their disease [10, 15].

#### Illness Perceptions

The second part of the questionnaire is built-up of 38 statements concerning views on the illness under study as evaluated on a five-point scale (strongly disagree to strongly agree) that together are divided into seven subscales, including:

1. *Timeline acute/chronic* Includes six questions that assess the timeline of the disease. Do the patients regard their illness as an acute/temporary condition, or do they believe their condition to be more permanent?
2. *Timeline cyclical* Includes four questions that differentiate between daily changes in symptoms of the disease and constantly present symptoms.
3. *Consequences* Includes six questions on the effect and consequences of the condition on patients’ daily lives.
4. *Emotional representations* Includes six questions that aim at quantifying the emotional response (anxiety, anger, depression, etc.) of a patient to his or her condition.
5. *Personal control* The combined score of six questions that represent the extent to which patients perceive having control over their illness.
6. *Treatment control* Five questions about whether a patient perceives the illness to be under control with (current) treatment.
7. *Illness coherence* Five questions on the patients’ personal understanding of their disease.

#### Causal Mechanisms

This part covers any mechanisms that patients may believe to be responsible for their disease and consists of 18 statements regarding perceived causes on a scale ranging from strongly agree to strongly disagree. Using a principal component analysis with varimax rotation, the causal items are clustered into variables with shared variance [10, 16]. These variables represent the causes of their disease from the patients’ perspective. Higher scores on the subscales indicate stronger beliefs in those attributions causing the disease.

### Principal Component Analysis

The factor analysis generated four factors, accounting for 71% of the total variance. Two factors accounted for the largest part if the variance: psychosocial attributions (accounting for 30% of the variance) and environmental factors (accounting for 23% of the variance), with a Cronbach’s α above the lower limit of 0.3 (0.937 and 0.865, respectively). Examples of the factor ‘psychosocial attributions’ include that FD is caused by daily stress or by a patients’ behavior; for the factor ‘environment’ that the disease is caused by heredity or a history of poor medical care. The other two factors, ‘accident’ and ‘chance’ were excluded from further analysis because of a low Cronbach’s α.

### Interpretation of the IPQ-R Outcomes

To put the outcomes of IPQ-R scores of FD patients in perspective, we compared these with reference groups of patients with acute pain [10], chronic pain [10], acromegaly [17] and fibromyalgia [6, 17, 18]. Data on reference groups of patients with acute pain (less than 6 weeks) and chronic pain (longer than 3 months) used in this study, originated from the study used to develop the revised version of the IPQ-R [10]. We also chose to compare the illness perception outcomes of our patients with FD with outcomes from a study on patients with acromegaly (47 male and 34 female), as patients with this disorder have been shown to be self-conscious about their appearance, leading to psychological distress, disruptions in everyday life and eventually impairments in QoL [19]. Although acromegaly is not characterised by facial asymmetry, patients with acromegaly do share a number of similarities with patients with craniofacial FD and MAS, such as excessive GH production, chronic joint pain, and marked facial deformities, which provided the rationale for choosing patients with this disorder as reference group. Lastly, we compared the scores in our set of patients with those of a cohort of patients with fibromyalgia (FM) (47 female and four male), as this is also a chronic disease associated with (multifocal) musculoskeletal pain.

### Identification of Associated Factors for Illness Perceptions

IPQ-R domain scores were compared between the different types of FD (monostotic/polyostotic/MAS) because of the broad spectrum of clinical manifestations of FD between these subtypes. Scores were also compared between patients with and without craniofacial disease, as we hypothesized that patients with craniofacial disease might score worse on the emotional domains because these patients may perceive to attract more negative attention as a result of their visible craniofacial deformities [20].

Domain scores of the IPQ-R were evaluated for a possible relationship with two clinical parameters of disease extent and severity, including SBS and serum levels of FGF-23, to evaluate whether patients with more severe disease have different illness perceptions compared to those with milder forms of FD. We also assessed possible relationships between subscales of the SF-36 and different domains of the IPQ-R, for which the outcomes on the SF-36 in a previous study into QoL in persons with FD were used [3].

### Statistical Analysis

Statistical analysis was performed using SPSS for Windows, Version 23.0 (SPSS, Inc., Chicago, IL, USA). Results are presented as mean (± SD) or as median (intermediate range) and in case of categorical data as a percentage. Differences between FD subtypes (monostotic/polyostotic/MAS) were compared using ANOVA, with post hoc analysis undertaken where appropriate. Difference between craniofacial and non-craniofacial FD was assessed using the Student’s *T* test. The IPQ-R scores of our FD cohort were compared with reference cohorts with the use of the Student’s *T* test (with level of significance set at *p* ≤ 0.05). IPQ-R scores were checked for normal distribution and correlated accordingly with one another using either the Pearson’s Correlation Coefficient or the Spearman’s Rank Correlation Coefficient (with level of significance set at *p* < 0.01 to correct for multiple testing). Possible associations were assessed between the IPQ-R scores and the SF-36 scores (previously reported SF-36 outcomes in this cohort are included in supplementary Table 1).

## Results

### Patient Characteristics (Table 1)

Out of a total of 138 eligible patients who were invited to participate in the study, two patients refused to take part, eight patients did not participate due to non-FD-related comorbidities (e.g., colon cancer, multiple sclerosis, osteosarcoma (*n* = 2), chondrosarcoma, severe rheumatoid arthritis, cardiac failure and significant learning disability) and 27 patients did not respond to our invitation. There were no significant differences between responders and non-responders except for gender; significantly more women completed the questionnaires (*p* = 0.039). Response rate was 70.3% with a total of 97 patients, 65% women, completing the questionnaires. Median age at diagnosis of FD was 29 years (range 1–68) and median age at completion of the questionnaires was 46 years (range 16–80). Level of education was low in 10 patients, medium in 24 patients, high in 46 patients and unknown in 17 patients. Sixty-two patients (64%) had monostotic FD, 26 (27%) had polyostotic FD, 9 (9%) had MAS and 5 (5%) had intramuscular myxomas in the context of Mazabraud’s syndrome. The craniofacial bones were affected in 23 patients (23%) and mean SBS was 8.68 (± 12.40 SD). SBS (*p* < 0.001), average FGF-23 (*p* = 0.002), a history of at least one fracture (p = 0.001) and of surgical interventions (*p* < 0.001) significantly differed between FD subtypes (monostotic/polyostotic/McCune–Albright syndrome) with increasingly higher SBS, FGF-23 levels and fracture and surgical intervention rates in the more severe subtypes. Median duration of follow-up was 12 years (range 0–62 years).

**Table 1:** Patient characteristics

	(*n* = 97)
Gender (male/female)	34/63
Age	46 (16–80)
Educational Level	Low 10 (10%)
	Medium 24 (25%)
	High 46 (47%)
	Unknown 17 (18%)
Type of FD	Monostotic 62 (64%)
	Polyostotic 26 (27%)
	McCune–Albright 9 (9%)
	Mazabraud 5 (5%)
Follow-up (years)	12 (0–62)

Data are median (range) or number and percentage

### Illness Perceptions in FD Patients Compared to Reference Groups (Table 2)

Compared to patients with acute pain, patients with FD were more aware of the chronicity of their disease (*p* < 0.001), perceived more fluctuations (*p* < 0.001) and had a better personal understanding of their disease (*p* < 0.001). However, patients with FD experienced less personal control (*p* < 0.001) and less treatment control (*p* < 0.001) of their illness.

**Table 2:** Comparison of IPQ-R scores between fibrous dysplasia patients and different patient groups

IPQ-R	Fibrous dysplasia *n* = 98	Acute pain [10] *n* = 35	Chronic pain [11] *n* = 63	Fibromyalgia [18] *n* = 51	Acromegaly [17] *n* = 81
Identity	3.5 (3)	2.8 (2)	6.2 (3)^β^	5.5 (2)^β^	2.5 (2)
Timeline acute/chronic	25.1 (5)	13.4 (5)^β^	23.1 (4)^α^	25.4 (4)	22.9 (6)^α^
Timeline cyclical	12.1 (3)	9.4 (3)^β^	12.9 (4)	15.0 (3)^β^	10.1 (4)^β^
Consequences	16.5 (6)	14.2 (4)	23.5 (4)^β^	19.3 (4)^α^	16.9 (5)
Personal control	17.3 (4)	22.9 (4)^β^	18.4 (4)	19.5 (4)^α^	17.5 (5)
Treatment control	15.5 (4)	19.4 (3)^β^	14.2 (3)	15.7 (3)	18.1 (3)^β^
Illness coherence	15.9 (3)	9.3 (3)^β^	13.4 (5)^β^	15.9 (3)	17.5 (3)^β^
Emotional representations	14.7 (5)	16.1 (4)	19.8 (4)^β^	16.2 (5)	12.6 (4)^α^
Psychological attributions (score range 8–29)	13.2 (6)	NA	NA	NA	NA
Environmental (risk) factors (score range 7–23)	12.2 (5)	NA	NA	NA	NA

Data are mean (SD)

*NA* not applicable

^α^ *p* < 0.01 compared with patients with fibrous dysplasia

^β^ *p* < 0.001 compared with patients with fibrous dysplasia

Compared to patients with chronic pain, patients with FD attributed less of their symptoms to their disease (*p* < 0.001) and perceived more chronicity (*p* < 0.01) and less consequences (*p* < 0.001) of their illness, had a better personal understanding of their disease (*p* < 0.001) and less emotional representations (*p* < 0.001).

Compared to patients with fibromyalgia, patients with FD attributed fewer symptoms to their disease (*p* < 0.001), perceived fewer fluctuations (*p* < 0.001), perceived fewer consequences (*p* < 0.01) and perceived less personal control (*p* < 0.01).

Compared to patients with acromegaly, patients with FD perceived more chronicity (*p* < 0.01) and more fluctuations of their symptoms (*p* < 0.001) and had less control over their treatment (*p* < 0.001). They also had worse personal understanding of their disease (*p* < 0.001) and perceived more emotional representations (*p* < 0.01).

### Illness Perceptions in Different Subtypes of Fibrous Dysplasia (Table 3)

There was a significant difference between subtypes of FD for: identity (*p* = 0.006), timeline acute/chronic (*p* = 0.002) and consequences (*p* < 0.001). Patients with MAS consistently demonstrated more negative illness perceptions in these domains compared to patients with polyostotic FD without endocrinopathies. Similarly, patients with polyostotic FD scored higher in these domains compared to patients with monostotic FD, illustrating that patients diagnosed with more severe subtypes of FD more often attribute their symptoms to FD, regularly experiencing their disease as chronic, reporting more consequences of their illness. There were no significant differences in other subscales of the IPQ-R. Patients with craniofacial FD scored significantly higher in the consequence domains (18.8 ± 5.7SD vs. 15.6 ± 5.6SD; *p* = 0.022) but this difference disappeared after correction for the presence of MAS. There were no significant differences between patients with and without craniofacial disease in other subscales of the IPQ-R.

**Table 3:** Comparison of IPQ-R scores between subgroups of fibrous dysplasia

IPQ-R domain	Monostotic *n* = 62	Polyostotic *n* = 26	McCune–Albright *n* = 9	Craniofacial *n* = 23	Not craniofacial *n* = 74
Identity	2.8 (3)^γ^	4.4 (4)	6.2 (6)^α^	4.6 (4)	3.2 (3)
Timeline acute/chronic	23.9 (5)^β^	27.4 (4)^α^	28.3 (2)	25.5 (5)	25.1 (5)
Timeline cyclical	11.7 (4)	13.3 (5)	13.3 (5)	12.2 (3)	12.2 (4)
Consequences	14.6 (6)^β,γ^	19.0 (4)^α^	21.4 (6)^α^	18.8 (6)^α^	15.6 (6)^α^
Personal control	17.3 (4)	17.5 (4)	16.3 (4)	16.7 (4)	17.5 (4)
Treatment control	15.6 (4)	15.7 (3)	14.1 (4)	16.5 (4)	15.2 (3)
Illness coherence	15.7 (3)	16.3 (2)	16.3 (3)	15.5 (3)	16.0 (3)
Emotional representations	14.3 (6)	15.8 (4)	13.9 (4)	15.3 (5)	14.5 (5)
Psychological attributions (score range 8–29)	14.1 (6)	12.0 (5)	9.4 (3)	11.4 (5)	13.7 (6)
Environmental (risk) factors (score range 7–23)	12.8 (5)	11.8 (5)	8.6 (3)	11.4 (5)	12.5 (5)

Data are mean (SD)

^α^ *p* < 0.05 compared monostotic

^β^ *p* < 0.05 compared polyostotic

^γ^ *p* < 0.05 compared with McCune-Albright

### Associated factors for Illness Perceptions (Table 4)

High skeletal burden scores as an indicator of severe disease were found to correlate with perceiving more consequences (*R* = −0.35; *p* = 0.003) and with more psychological attribution domains (*R* = 0.312; *p* = 0.010) of the IPQ-R. High average serum concentrations of FGF-23 correlated with attributing more complaints to FD (identity) (*R* = −0.389; *p* = 0.004) and perceiving more consequences of their illness (*R* = −0.37; *p* = 0.007).

**Table 4:** Associated factors for illness perceptions in fibrous dysplasia

IPQ-R domain	SBS		FGF-23	
	SRCC	Sig.	SRCC	Sig.
Identity	−0.25	0.039	−0.389	0.004
Timeline (acute/chronic)	−0.27	0.027	−0.03	0.822
Timeline (cyclical)	−0.04	0.751	−0.14	0.310
Consequences	−0.35	0.003	−0.37	0.007
Personal control	0.07	0.582	−0.06	0.650
Treatment control	0.06	0.625	−0.04	0.804
Illness coherence	−0.19	0.115	−0.18	0.191
Emotional representations	−0.05	0.716	−0.20	0.910
Psychological attributions	0.312	0.010	0.070	0.620
Environmental (risk) factors	0.268	0.027	0.199	0.158

Attributing factors for illness perceptions in fibrous dysplasia with significance set at *p* = 0.01

*SRCC* Spearman’s rank correlation coefficient

### Relationship Between Illness Perceptions and QoL (Table 5)

Attributing more symptoms to the disease was associated with perceiving more limitations in the domain’s physical functioning (*p* < 0.001), physical role (*p* < 0.001), bodily pain (*p* < 0.001), general health (*p* < 0.001), social functioning (*p* < 0.001) and mental health (*p* < 0.001). A more chronic experience of FD was associated with perceiving more impairments in physical function (*p* = 0.005) and more bodily pain (*p* < 0.001). Experiencing symptoms of FD more chronically throughout the day was associated with more impairments in physical function (*p* = 0.005), social function (*p* = 0.01) and more bodily pain (*p* = 0.002). Both experiencing more consequences of FD and having more emotional representations of FD were associated with more impairments in all domains of the SF-36. Lastly, perceiving less treatment control was associated with more impairments in general health (*p* < 0.001) and vitality (*p* < 0.001). There were no significant correlations found for other IPQ-R dimensions.

**Table 5:** Correlations between SF-36 and IPQ-R domains in fibrous dysplasia

SF-36 domain	Identity		Timeline (acute/chronic)		Timeline (Cyclical)		Consequences		Personal Control		Treatment control		Illness Coherence		Emotional representations		Psychological attributions		Environmental (risk) factors	
	*R*	Sig.	*R*	Sig.	*R*	Sig.	*R*	Sig.	*R*	Sig.	*R*	Sig.	*R*	Sig.	*R*	Sig.	*R*	Sig.	*R*	Sig.
Physical function	−0.52	<0.001	−0.30	0.005	−0.28	0.010	−0.58	<0.001	−0.13	0.246	0.22	0.460	−0.14	0.202	−0.32	0.003	−0.11	0.316	−0.06	0.612
Role physical function	−0.37	<0.001	−0.13	0.255	−0.21	0.053	−0.58	<0.001	−0.03	0.789	0.14	0.191	−0.03	0.809	−0.39	<0.001	−0.14	0.197	−0.06	0.586
Bodily pain	−0.58	<0.001	−0.40	<0.001	−0.32	0.002	−0.38	<0.001	0.01	0.935	0.25	0.015	0.11	0.288	−0.33	<0.001	−0.01	0.929	−0.02	0.887
General health	−0.39	<0.001	−0.18	0.093	−0.16	0.125	−0.46	<0.001	0.06	0.592	0.36	<0.001	0.13	0.223	−0.37	<0.001	−0.29	0.006	−0.20	0.053
Vitality	−0.25	0.015	−0.14	0.194	−0.04	0.688	−0.37	<0.001	0.22	0.037	0.47	<0.001	0.11	0.320	−0.38	<0.001	−0.23	0.027	−0.10	0.340
Social function	−0.50	<0.001	−0.23	0.027	−0.28	0.010	−0.56	<0.001	−0.04	0.700	0.25	0.017	0.10	0.367	−0.43	<0.001	−0.17	0.111	−0.09	0.372
Role emotional	−0.26	0.018	0.05	0.641	−0.20	0.068	−0.36	0.001	−0.12	0.289	0.03	0.804	−0.06	0.592	−0.33	0.002	−0.16	0.146	−0.05	0.645
Mental health	−0.37	<0.001	−0.12	0.299	−0.11	0.329	−0.33	0.002	0.06	0.624	0.26	0.017	0.17	0.121	−0.43	<0.001	−0.22	0.048	−0.12	0.303

Correlations between SF-36 and IPQ-R domains with level of significance set at *p* = 0.01

*PF* physical function, *RP* role physical, *BP* bodily pain, *GH* general health, *VT* vitality, *SF* social function, *RE* role emotional, *MH* mental health

## Discussion

Our data indicate that illness perceptions significantly differ between patients with FD and patients with a number of other conditions associated with acute or chronic pain. Patients with FD report more negative illness perceptions than patients with acromegaly, but more positive illness perceptions compared to patients with fibromyalgia.

In comparison with the reference groups, personal and treatment control were the most prevalent negative illness perceptions in FD patients, indicating that these patients experience very little control over the course and treatment of their condition. This can partially be attributed to the chronic character of FD, since chronicity of the disease was perceived to be higher in patients with FD compared patients with either acute or chronic pain and those with acromegaly, possibly as a reflection of current limitations in treating the disease. There is no cure for FD and current treatment modalities indeed fail to provide a long-term solution for the symptoms of FD, although surgical interventions or medical therapy are to some extent able to decrease pain and improve function. A significant proportion of these patients need multiple surgical interventions or continuous use of anti-resorptive agents due to persistent symptoms of continuous pain as well as recurrent fractures [21–23]. Further development of effective medical treatments could help to increase the faith that patients would have in their control over their disease. Developing disease-specific self-management skills could possibly boost personal control over the disease, for example with appropriate mobilization aids or more specifically with self-management training, aimed at exploring and changing the illness perceptions and therefore coping strategies of the individual patient [4, 24]. In a biopsychosocial approach, this would mean that negative thinking about FD should be identified early to initiate an intervention aiming at better adaption to FD and more adaptive expectations [9]. In FD, this could for example mean adaption of unrealistic ideas about mobilizing or actively participating in sports due to an increased fracture risk. Once identified, a possible intervention to address these negative perceptions would be to stimulate patients to develop a personal plan on mobilizing and participating in sports in a fashion that minimizes the risk of fracturing a bone with fibrous dysplasia. Developing a personal plan of action has previously been used to adequately address illness perceptions in persons with other conditions [9, 25]. As a result of this personal plan, these patients might increase their physical activity and are likely to similarly increase their appreciating of their QoL.

Data from this study also show that patients with the more severe types of FD attribute a greater part of their symptoms to their illness, experience their FD as more chronic and are perceiving more consequences in their daily life. These findings are in line with the more severely impaired QoL in these severe subtypes and are further underlined by the association of high SBS and serum levels of FGF-23, both indicators of more severe disease, with negative illness perceptions of the consequences of FD. [3, 13, 14] Interestingly, several studies reporting on pain deriving from bony lesions in patients with fibrous dysplasia fail to find an association between pain severity and SBS, consistently describing a wide variation in pain symptoms between patients with FD who are similarly affected. [3, 26] However, our data show that increased perception of bodily pain, as assessed by the SF-36 questionnaire, is associated with attributing more symptoms to the disease, a more chronic experience of FD, experiencing symptoms of FD more chronically throughout the day, experiencing more consequences of FD and having more emotional representations of FD. The precise pathogenic mechanism underlying the development of pain in patients with FD remains to date elusive [27]. Although following a variable pattern across the wide clinical spectrum of FD, the presence of pain appears to predict multiple negative illness perceptions, with pain being probably an important factor contributing to impairments in quality of life in these patients.

As previously shown in patients with other (endocrinological) disorders such as for example Cushing’s syndrome and acromegaly, the current study demonstrates that negative illness perceptions are associated with impairments in many QoL domains as assessed by the SF-36 questionnaire [4, 17, 28]. This especially includes the illness perception domains of identity, timeline, consequences and emotional representations. It is, therefore, plausible that addressing these perceptions might prove to be a useful tool in the management of patients with FD. Current treatment modalities are unable to fully control the symptoms of FD, particularly in the more severe type of the disease. Psychological support aiming at minimizing negative illness perceptions may thus go a long way to improve QoL in the long term in these patients. This is particularly important, as there is no cure for the disease. Cognitive behavioral therapy, self-management training or information on the possible negative effects of FD applied at an early stage in the course of the disease may, therefore, improve maladaptive illness perceptions, probably eventually leading to improved function and QoL in patients with FD [4, 7].

Our study has strengths as well as limitations. Despite the fact that we were able to investigate both QoL and illness perceptions in a relatively large cohort of patients with FD, we appreciate that especially the group of MAS patients, and therefore the significant differences found in this group, are underpowered and should be interpreted with care. This limitation is generally shared with all rare and heterogeneous diseases and in future research this could possibly be addressed by extending our levels of collaboration between international centers in these types of disorders. However, it is likely that the differences that we found between the subtypes of FD will be even more evident in stronger powered studies. In addition, we were able to get a good representation of our cohort of FD patients with a response rate of 70.3%. The other main limitation of our study lies in its cross-sectional design, with a single-time-point measurement of both illness perceptions and QoL, precluding any statements about cause and effect, therefore. It would be interesting to analyze the course of illness perceptions and QoL over time and especially the effect of self-management on the course of FD. Despite these limitations, we believe that our current data holds important leads for physicians involved in medical care for patients with FD, in understanding the impairments of FD in individual patients and the perceptions of these patients regarding their disease.

In conclusion, this first study on illness perceptions in patients with FD demonstrates that illness perceptions are affected throughout the wide spectrum of fibrous dysplasia and that these perceptions are associated with impairments in quality of life. Severity of disease as expressed by high skeletal burden scores and increased serum levels of FGF-23 were associated with these maladaptive illness perceptions. Altering unhelpful illness perceptions in these patients may represent a promising tool in the management of patients with FD, particularly in the more severely affected patient by helping coping behavior and improving quality of life in FD.

## Electronic Supplementary Material

Below is the link to the electronic supplementary material.

Supplementary material 1 (DOCX 20 kb)
