# Supplementary material for: Illness Perceptions are Associated with Quality of Life in Patients with Fibrous Dysplasia
Source: Calcif Tissue Int. 2017 Oct 11;102(1):23–31. doi: 10.1007/s00223-017-0329-5 (PMC5760610; doi:10.1007/s00223-017-0329-5)
Supplement: Supplementary file 1 — Supplementary material 1 (DOCX 20 kb) [file 223_2017_329_MOESM1_ESM.docx]

| **Supplementary Table 1. Results of SF-36 in patients with fibrous dysplasia (1)** | | | | |
| --- | --- | --- | --- | --- |
| **SF-36** | **Monostotic, n=62** | **Polyostotic, n=26** | **McCune-Albright,  n=9** | **General population, n=97** |
| Physical Function | 83.1 (20) | 63.9 (26)^β^ | 51.1 (34)^β^ | 74.5 (26) |
| Role Physical | 71.7 (39) | 63 (44) | 44.4 (39)^β^ | 66.5 (42) |
| Bodily Pain | 73.9 (25) | 60.5 (27)^β^ | 57.1 (25)^α^ | 68.7 (26) |
| General Health | 63.1 (23)^β^ | 52 (22)^β^ | 55.6 (30)^α^ | 59.4 (24) |
| Vitality | 62.2 (18)^α^ | 59.4 (19)^α^ | 57.2 (16) | 60.6 (18) |
| Social Function | 82.3 (21) | 71.5 (28)^β^ | 61.1 (33)^β^ | 77.4 (25) |
| Role Emotional | 84.3 (31) | 87.5 (29) | 88.9 (24) | 85.5 (29) |
| Mental Health | 73.6 (18) | 77.7 (14) | 75.6 (10) | 75 (16) |
| Data are mean (SD). |  |  |  |  |
| ^α^ P<0.05 compared to the general population | |  |  |  |
| ^β^ P<0.01 compared to the general population | |  |  |  |

1. Majoor BCJ, Andela CD, Bruggemann J, et al. Determinants of impaired quality of life in patients with fibrous dysplasia. Orphanet journal of rare diseases. 2017;12(1):80. doi:10.1186/s13023-017-0629-x
